# Report of the Committee of the American Society of Dental Surgeons, on the Propriety of Rescinding the Amalgam Pledge

**Published:** 1850-10

**Authors:** Elisha Townsend, Joseph H. Foster

**Affiliations:** Committee.; Committee.


### ARTICLE XI.

*Report of the Committee of the American Society of Dental Surgeons, on the Propriety of Rescinding the Amalgam Pledge.*

Mr. President:—The committee to whom the Society, at its last adjourned meeting, held in Baltimore, referred the paper read before them, on the propriety of rescinding the amalgam pledge, respectfully report: That they have given to the matters involved, the earnest attention which their importance demands, and beg leave to submit the result of their deliberations to the judgment and disposal of the Society. Your committee having agreed to recommend the rescission of the resolution or resolutions, under which subscription to the protest and pledge is made a condition of membership, respectfully offer a concise view of the considerations which have led them to this conclusion.

The organization of the American Society of Dental Surgeons was grounded, doubtless, upon all those views of sound policy, and urged by all those impulses of professional fraternity, which induce similar co-operative unions in other liberal branches of art and science.

The associative sentiment which prompts to the combined order as the method of great enterprises, springs from one of the noblest instincts of our nature; and its necessity is felt, and its advantages are illustrated, in proportion to the rank and value of the undertaking in which it is enlisted. But, in addition to all this, the special exigencies of the day, temporary in their own nature, but of critical importance to our profession, in the forming stage of its history—were felt to be the immediately pressing occasion for a national organization. All the necessities of fraternization were acknowledged, and all its general usefulness was appreciated, but the main interest of the emergency was the creation of a conventional tribunal, with its incident authority, to assume the responsibilities of the profession, and to establish and vindicate its corporate character. The formation of this Society was the earliest general attempt to run a clear line of discrimination between the man of competent attainments and the impudent empiric; not by arrogating to its own membership the exclusive claim of regular professional standing, but by erecting a general and authoritative standard, by which the claims of our department of surgical art might be formally asserted, and respectable qualifications be better distinguished from unwarranted pretension. Such motive was justifiable as a matter of individual self-respect and self-defence; but it also deserved the credit of a generous regard for the honor of the whole body of worthy practitioners. In the preamble to the constitution, this object is expressed in the form of a summary of the inducements to the organization; after enumerating important general aims, the article concludes thus: "In fine, to give character and respectability to the profession, by establishing a line of distinction between the truly meritorious and skilful, and such as riot in the ill gotten fruit of unblushing impudence and empiricism."

For all the reasons that men of honor and probity, bearing a common professional appellation, should associate in order to establish a standard and a reputation for their profession, and at the same time apply the corrective of authoritative opinion to abuses over which they had no other power, the dentists of the United States, eleven years ago, organized the American Society; and among the mischiefs calling for remedy, at their hands, a very conspicuous one was the business of amalgam or mineral paste fillings. Under seven different names, at least, but with no real change in the essentially mischievous ingredients, the mineral paste was used by all sorts of pretenders and impostors as a cheap, agreeable and safe substitute for the nobler metals in the filling of teeth and their fangs.

In the free and easy morality of the Greek mythology, Mercury was assigned the post of patron deity of thieves; it was a capital hit—a regular piece of oracular inspiration, that gave his name to the metal whose facility of use in all sorts of quackery, well entitles it to the honor of his slippery godship's cognomen, as the great facility of motion to which it is usually ascribed. General therapeutics had for centuries afforded large play ground for this mountebank miracle-worker; and dentistry, by a sort of inheritance, was destined to sustain an assault in its turn, but thanks to the giant energy of the youngest born of the healing arts, even in its cradle, it was able to strangle the snake, and break the wand of the enchanter.

Before the formation of the Society, the Crawcour, and soon after the Malan, imposture had their run, and such was the odious notoriety of their practice, that the question of amalgam filling was as familiar, at least, if not as clearly understood, in the mind of the community at large, as in that of the profession; and such generally were the circumstances and the excitement of the occasion, that respectable men felt the necessity of distinguishing, in the most explicit way, the practice of the regular profession, from all possibility of identification with such atrocious empiricism. An abuse of the public trust, and a violation of the principles of our art so gross as *these* charlatans had practiced, of course, men of *reputation* had no necessity for themselves to disavow. But there were other possibilities of mischief in this matter, not so *obvious*, nor so amenable to public reprehension, which fell properly within the care and correction of the faculty itself, and to which the Society felt bound under the circumstances to apply a remedy. There was something in this sin against science so easily besetting to incautious, inexperienced and imperfectly qualified practitioners, that they were but too likely to stretch the probabilities in its favor, and to practice it to the detriment of all good dentistry, and the general discredit of the professional name. Moreover, the great body of dentists then fully agreed in the opinion that any and every use of the mineral pastes was mal-practice, and reprehensible on the triple ground of fraud upon the patient's purse, danger to his health, and injury to the interests and advancement of the profession.

The Society, therefore, under the impulse of self-preservation, and in obedience, also, to a public duty, repudiated and denounced the practice, and proceeding, as it believed the case required, from a declaration of opinion, which met with no formally displayed opposition to its enforcement, at last, in 1845, exacted the protest and pledge from all within its jurisdiction. The stringent measure of compelling conformity of practice by the penalty of expulsion in a matter of science, which, though almost universally assented to, still lay in the naturally free domain of opinion, and was not a subject of explicit constitutional or conventional compact, is open, certainly, to general exceptions, and must bear the onus of defending itself against the prima facia violation of permanent, uniform and universal principles. The argument for liberty of opinion and correspondent liberty of action, is obvious aud conclusive, even to demonstration, it is equal to intuition in clearness and authority ; and the exceptional abridgement or suspension of the right must be able to plead such exigencies as in all affairs of life are allowed temporarily to over-ride the settled constitution of things ; even as martial law, military conscription, police levies, and the irresistible concomitants of flood, fire and pestilence, in their several ways, take authority from their sheer necessity.

Our profession, at the date of these events, was young ; it was very imperfectly organised ; it had, as yet, made no such lodgement in public confidence, as might shelter it from plausible charges of empiricism ; it was crippled by its own incapacities, as well as burdened with the weight of injurious misconstruction, and only too large a proportion of those who bore the name, were raw and ignorant adventurers, or worse, reckless fugitives from other professions and avocations, for whose duties they were, on trial, found to be incompetent: indeed, even a large number of the tolerably honest and tolerably successful knew but too little of the science, and felt too little of its enthusiasm and of its responsibilities, to form a safe judgment in the premises, or to follow a sound practice against strong temptation, if they even obtained some glimpses of the truth. To all this, add the seductive facilities of the operation ; the falsely flattering exemption from the disagreeable of operating and enduring to both dentist and patient ; the cheapness of material, and above all, its plastic property, doing its own drilling, fitting and hardening, and there is enough to explain and to warrant the most *determined* and decisive action on the part of those who had married the profession for better for worse, and must stand or fall with its fame and fortunes.

Moreover, the curative treatment of diseased teeth, being at once the most difficult and the most important branch of dental surgery, was then, as it is yet, and must ever be, the one in which the highest art has most to achieve, and in which it is most exposed to the indirect practices of ignorance, indolence and dishonesty, within and without the regular ranks. Here, just here, the Society felt, in all its instincts, was the post of danger, as it was the post of honor ; and it may well be excused to a generous *esprit du corps*, and an honorable sense of public duty, if, either in form, manner, spirit or effect, it transcended, in some slight degree, those nice limits between expediency and error, which are not only difficult to determine, but, in fact, shift their position with circumstances, and even with the opinions and caprices of the parties engaged in them. In every branch of science and art, in every department of practical affairs, there are representative men—men who, without usurpation, without any formal design, almost without even the consciousness of the fact, virtually legislate for their class. They are rulers and patterns by the highest right, the divine right of fitness. In emergencies these men are sure to take the direction and the responsibility of affairs. They lead revolutions as provisional governments and committees of public safety, and they rule by the inspiration of the occasion. Precedents, which are often mistaken for principles, do not serve the turn; the general welfare demands the protection of a higher rule, better adjusted to the hour and the exigency. The criterion by which such actions are to be judged, is the issue and the end which is attained. If the affair is conducted in all respects to a happy result, securing permanent good, with no abatement of enduring evil, it stands justified, and the agents are vindicated. It is not questioned, and it need not be denied, that after any successful struggle, with *all* the facts in full view, a critic may be able to say where a little ammunition might have been saved, and where a little blood might have been spared, without endangering the result. Nay, an objector, who takes no other responsibility of an enterprise than that of finding fault, may be able to demonstrate, after the battle, just as he asserted before it, what nice points of principle, and close calculations of propriety, were violated in the heat and hurry of the engagement, All these things are truly matters of regret, and may well check the exultations, although they cannot destroy the value of the victory. But *after* the battle, the only question remaining really worthy of general concern is, how the peace may be adjusted, and its great interests be best secured. The combatants must not indulge their spite by trampling on the dead and killing the *cripples*, after the conflict is fairly over; in other words, a high debate, made necessary on both sides by sentiments of honor and duty, must be protected from descending into the gossip of a personal wrangle, after the unhappy question of difference itself is settled. As a first step to a consummation so desirable, we propose the repeal of the resolutions which enforce the amalgam pledge, in the persuasion that it has fulfilled its office, and served its turn, and may therefore be honorably discharged from the service. It is now seven years since the Society, "unanimously resolved, that it regards the use of mineral pastes as stoppings for carious teeth as malpractice." In 1841 with like unanimity, it had declared that amalgams were hurtful to the teeth and every part of the mouth, and that gold could be used in every case where any form of filling fairly promised advantage, and at subsequent annual meetings it reiterated these sentiments, till finally in 1845 it resolved upon the expulsion of non-conformists, and in 1847 actually inflicted the penalty upon some of its members.

Testimony so uniform, continuing and unwavering, and action so rigidly conformable, and maintained at such expense of professional and personal attachments, as the censure and expulsion of many highly prized friends and brethren, is, we believe, enough for the end aimed at, enough for all the requirements of duty in the premises, and because enough, quite as much as we can constrain ourselves to do and endure in relation to it. Seven years bring great changes in the things around us, in ourselves, and in all our relations and attitudes to the world's movements. The condition of things which at the beginning of that period controlled our conduct, are all changed, and with them, the forms of duty and expediency which the most rigid principles must take from varying circumstances.

A young and rapidly rising profession like ours is almost transformed, as the human body is popularly supposed to be in the course of a septennial cycle. It maintains its identity indeed, as the infant still lives in the man, but its position to passing interests, its strifes and dangers, and its duties in relation to them are no longer the same, because it has become wholly a matter of individual responsibility.

The reputation of the profession has been steadily growing during the interval through which this strife prevailed, and has fairly settled into such established consistency as serves, now, quietly to avert impeachment of empiricism, that ten years ago would have involved the fraternity in the judgment of but too many of the community for our interests and honor, however unjust and contemptible for palpable untruth and absurdity.

Dentistry now, like the practice of medicine, may safely trust its general character to the common caution and prudence which legitimate responsibility ordinarily requires. No doubt many causes have operated together to produce the present confidence in the integrity of our profession, felt by the public; but it is very safe to infer, that the nice sense of professional honor, and the jealous strictness of discipline which the national society has so long manifested in its own unfearing and unflinching way, did much to impress upon the public mind our own estimation of our duties and dignity, and served, at the the same time, to re-impress and deepen the sentiment upon ourselves and the entire profession, as well those without as those within the Society lines. If it be true that even the collision of opposite errors often elicits truth, then the earnest conflict of a high-hearted sentiment, pursuing right ends by direct means, against a wrong which rested its defence wholly in exemption from conventional control, must have contributed eminently to the fortunate issue which has resulted. For, whether the Society was right, as it honestly believed, in all its ends and means, or unfortunate in some circumstances of its method, it cannot be doubted, we think, that the exhibition and vindication of a corporate spirit, a professional conscience, a jealous regard for the public welfare, and a scrupulous subjection of its own membership to limitations and restraints, up to the point where honor and duty meet and may not pass each other, must have contributed nobly, in every way, to that enhanced regard which the community has learned to accord to the profession of dental surgery. Moreover, the profession itself has been advancing all the while, in improved views and methods of treating diseased teeth, and in juster apprehensions of the paramount importance of this branch of practice; and as it would be unphilosophical to exclude any cause actually in operation from a share in the effect, the pledge and protest may vindicate their claim to a portion, probably a large portion, of that excellent result which has accrued in the matter upon which their action was directed; and in the same ratio that the faculty has itself advanced, the public has learned to distinguish mere pretension from real ability. It is ever so. The men of any calling, which rests in reputation, need but to be true to themselves and to principle, during their term of probation, and they will mould the mind of the public to the impress of their own. But, however arrived at, and by whatever means achieved, your committee believe that the time and the conditions have come, when this Society is no longer responsible to itself, nor held so to the community, for any of the mischiefs of amalgam filling in the practice of its members or others. And having no other rightful authority in the premises, than that which arises out of necessary self-defence, and the duty to the public which is involved in its societary function; the obligation of official action ceases with the necessity, and the expediency of a crisis which has passed, gives way to the regularity of the general rule.

The Society certainly never meant, by the exaction of this pledge, to abridge the natural liberties of opinion, or to settle upon principle any question of science by force of a majority, or even by an unanimous vote ; *but* to protect itself against injuries, to forward the objects of its organization, and to promote the corporate welfare.

While, therefore, we would intimate no change of sentiment as to the subject matter of the protest, believing, as we do, the substitution of amalgam for gold to be malpractice, but would still most earnestly *advise that* total abstinence from the practice, which we have heretofore *enforced.* We recommend the repeal of all the resolutions in regard to amalgams, [enforced by penalties,] and submit the following resolution for adoption:

*Resolved*, That the several resolutions adopted by the American Society of Dental Surgeons, at their annual meetings held in 1845 and 1846, having the effect of enforcing subscription to the protest and pledge against the use of amalgams and mineral paste fillings for teeth, be and the same are hereby rescinded and repealed.

All of which is most respectfully submitted.

ELISHA TOWNSEND,<br>JOSEPH H. FOSTER, } *Committee.*

